# When and how should peritoneal endometriosis be operated on in order to improve fertility rates and symptoms? The experience and outcomes of nearly 100 cases

**DOI:** 10.1007/s00404-021-05971-6

**Published:** 2021-02-03

**Authors:** A. M. Dückelmann, E. Taube, E. Abesadze, V. Chiantera, J. Sehouli, S. Mechsner

**Affiliations:** 1 Department of Gynecology, Charité-Universitätsmedizin BerlinVirchow Klinikum, Augustenburger Platz 1, 13353 Berlin, Germany; 2 Department of Pathology, Charité-Universitätsmedizin Berlin, Charitéplatz 1, 10117 Berlin, Germany; 3 Department of Gynecology, Vivantes Clinic Berlin Hellersdorf, Myslowitzerstr. 45, 12621 Berlin, Germany; 4 Department of Gynecologic Oncology, University of Palermo, Piazza Marina, 61, 90133 Palermo, Italy

**Keywords:** Peritoneal endometriosis, Peritonectomy, Laparoscopic surgery, Pelvic pain, Infertility

## Abstract

**Purpose:**

To analyze the follow-up results of patients suffering from symptomatic early-stage endometriosis after a consistent laparoscopic peritoneal stripping of the altered peritoneum (peritoneal endometriosis and surrounding inflamed tissue) was performed. This type of endometriosis is resistant to medical therapy and/or impairs fertility.

**Methods:**

Using our prospectively maintained database, we were able to identify all symptomatic women with the suspicion of only peritoneal endometriosis who underwent laparoscopy at our endometriosis center over a period of 5 years. All procedures were carried out in a standardized fashion by one single surgeon, who is highly experienced in minimal invasive surgery, and included a suspended hormonal pretreatment for 2 months. Postoperative outcomes including complications, fertility and recurrence rates were analysed.

**Results:**

Laparoscopic peritonectomy was performed on 94 women. Follow-up data were available in 87% of these cases. At the time of surgery, almost all patients tested showed signs of stage I or II endometriosis (44.7 and 48.9%, respectively). More than three-quarters of the women reported pain relief, inter alia, due to the post-surgical hormonal therapy. About one-third of the patients wanted to have children after the procedure. 62% of them became pregnant and the majority did so without the need for assisted reproductive therapy. In seven women a re-operation was performed.

**Conclusion:**

According to our data, a consistent excision of altered peritoneum followed by adjuvant hormonal therapy and multimodal concepts results in better outcomes for the patient, particularly in regards to pregnancy and recurrence rates.

## Introduction

Endometriosis is a benign chronic inflammatory disease affecting millions of women worldwide during their reproductive years [1, 2]. The pathogenesis is still under debate, however, most likely, stem cell such as cells from retrograde menstruation adhere to the peritoneal surface and develop into peritoneal endometriotic lesions [3, 4]. Apart from the establishment of such ectopic lesions, numerous and brisk immune cell infiltrates were found within the microenvironment of these lesions, indicating acute immunological reactions [5, 6]. Both pathways are linked to each other. Macrophages are in the peritoneal fluid as well as in the peritoneal lesions and in the unaffected peritoneum from women with endometriosis, secreting a variety of pro-inflammatory cytokines and chemokines in the peritoneal fluid [7, 8]. Neurotrophins and neuronal guidance molecules and their receptors are most highly expressed in the glands of endometriotic peritoneal lesions [9]. The release of nerve growth factors leads to changes in the peritoneal innervation [8]. This includes the hyperinnervation of sensory nerve fibers and the hypoinnervation of sympathetic nerve fibers with an imbalance of pro- and anti-inflammatory neurotransmitters [10, 11]. As more nerve fibres were found in the areas where increased numbers of macrophages were identified, the density of macrophages seems to correlate with the number of nerve fibres, which in turn correlates with the development of endometriosis-related symptoms [8, 12]. Endometriosis-associated immune cell infiltrates might be a trigger for a neurogenic inflammatory reaction and a critical point where cyclical pain becomes acyclical pelvic pain [10, 11, 13–15].

Pain is the main symptom of endometriosis patients with a very heterogenous variation of several symptoms including dysmenorrhoea, cyclical and acyclical pelvic pain, dysuria, dyschezia, dyspareunia etc. [16]. These symptoms have a negative impact on the physical, mental, and social wellbeing of patients [17]. The severity of pain is independent of the stage/extent of the disease and the appearance and location of endometriosis deposits [18–22]. However, pain generation is very complex and the impact of peritoneal lesions on pain generation is difficult to understand and differentiate from symptoms caused by other kinds of illness, like adenomyosis, deep infiltrating lesions, endometrioma or adhesions [23]. Many patients suffer from a combined manifestation of lesions and present a combination of symptoms [23].

The first step in the treatment of patients with suspected endometriosis symptoms should be hormonal treatment. If this fails then surgery should be indicated as the next treatment option [24–28]. The therapeutic approach of peritoneal endometriosis worldwide is very heterogenous. Many patients receive laparoscopy for the diagnostic purpose only. So residual foci are left behind, which should be avoided as an outcome depending on the completeness of the surgical treatment. Compared to diagnostic laparoscopy only, the surgical management of mild endometriosis seems to be more effective in treating the symptoms of pain and improving the quality of life for women with endometriosis as well as improving their pregnancy rate [29–33].

In a randomized, placebo-controlled trial by Abbott et al. comparing immediate excision with delayed surgery on 39 women, of whom about 50% had rAFS stage I and II, surgery was associated with a 30% placebo response rate, not dependent on the severity of the disease. Approximately 20% of women did not report an improvement after surgery for endometriosis [34]. In another classical study by Sutton et al. on the same question (but on women with mostly stage I disease receiving laparoscopic ablation) the nonresponse rate was 38% [35]. Recently a review showed that many women only gain limited or intermittent benefits from long-term treatment [36].

As a consequence, early stage surgical intervention in endometriosis should be limited to patients with painful symptoms and contraindications or ones who show a poor response to medical therapies or in cases of subfertility [32]. Hormonal treatment as therapeutic attempt should always be performed before surgery, in particular in the absence of any sonographic evidence of endometriosis, to clarify the cause of pain and identify patients with symptomatic peritoneal endometriosis. The surgical approach should be reserved for clearly defined objectives: to reduce pain, increase patient’s pregnancy rate, exclude advanced stages of endometriosis or malignant adnexal masses and delay recurrence for as long as possible [37].

Aside from invasiveness, morbidity and complication risks, the recurrence of symptoms or lesions after surgery is highly concerning [38–40]*.* According to a review the 2 years recurrence rate is estimated to be 21.5% [41]. The association between disease relapse and rARSM stages is still under debate, recurrence is, however, markedly prevented by the administration of estroprogestins [41–45].

Sharp excision, bipolar diathermy and ablation by CO_2_-laser are the most common techniques in laparoscopic surgery for endometriosis. The question of which techniques should be preferred to manage superficial peritoneal disease has not yet been answered [46] (see Table 1).

**Table 1:** OP technique

	Kind of study	OP technique	Number of patients	Outcome
Healey et al. [73]	RCT	Excision vs ablation with CO_2_ laser	178	No significant difference at 12 months (improvement of patients’ symptoms in both arms). Trends in improvement of dyschezia and dyspareunia
Wright et al. (75)	Prospective randomized	Excision vs ablation	24	No significant difference (symptom relief at 6 months in both arms)
Radosa et al. (74)	Retrospective	Excision vs coagulation	79	Coagulation group better for dysmenorrhea and the number of recurrence with subsequent surgical intervention (2.8 vs 18.6%)
Riley [71]	RCT	Excision vs ablation by using an argon beam coagulator	73	Ablation group better for dyspareunia at 6 months only. No significant difference at 12 months

In practice, there is a tendency for gynecologic surgeons to prefer to perform ablation because it is considered easier. Theoretically, excision is advantageous because it ensures that the entire lesion or pathologic tissue is removed.

In our opinion, there is a lack of studies regarding the indication for surgery of peritoneal endometriotic lesions and the surgical procedure to treat them. This paper focuses on our experience and presents the follow-up results after laparoscopic peritoneal stripping of the altered peritoneum (peritoneal endometriosis and surrounding inflamed tissue).

## Materials and methods

We analysed our prospectively maintained database to identify all women who underwent laparoscopy at our endometriosis center from January 2014 to June 2019. Women with a sonographic exclusion of complex endometriosis manifestation and symptomatic endometriosis and/or impaired fertility older than 18 years were included. Indication for surgery was only given for typical symptoms of endometriosis after the failure of sufficient hormonal treatment (amenorrhoea > 6 months) with ongoing acyclical pelvic pain. The estimated endometriotic lesions were peritoneal lesions (with or without adenomyosis). Patients were not excluded if they had already been diagnosed with endometriosis. An initial survey of the pelvis was performed, and any patient found to have ovarian cysts or endometriomas, retrospectively, or any signs for deep infiltrating endometriosis, was excluded. Exclusion criteria included further intraoperative bilateral salpingo-oophorectomy and hysterectomy for adenomyosis. The goal was to concentrate on women with only peritoneal endometriosis.

The database contained all information about demographic and clinical characteristics, medical examination, imaging and surgical therapy. The preoperative pelvic pain severity was assessed by a 10-point visual analog scale (VAS) that was routinely performed at preoperative visits and covered different types of pain: dysmenorrhea, cyclic pain, complex chronic pain, dyspareunia, dysuria and dyschezia. VAS scores were a validated way to measure pain and used to measure overall pelvic pain as well as the different types of visceral pain [47]. We stated all clinically relevant symptoms with a score ≥ 5.

All procedures were carried out in a standardized fashion by one single surgeon, who is highly experienced in minimal invasive surgery for endometriosis. A suspended hormonal pretreatment for 2 months followed the surgery [48]. In all cases, a careful evaluation of the whole abdominal cavity was performed. The clinically suspected diagnosis was verified intraoperatively and all visible endometriosis implants and/or inflammatory altered peritoneum were radically excised (peritonectomy) including the removal of around two cm of the surrounding normal-appearing tissue (wide excision). For classification, we used the revised score of the American Society of Reproductive Medicine (rARSM) [49]. Excision was carried out by grasping the peritoneum with the endometriotic lesion, thus distancing it from the underlying tissue. Using laparoscopic scissors, the lesion along with a border of normal peritoneum was extracted [48]. We did not use barrier methods to prevent adhesions. Excised lesions were submitted for histological examination to confirm the diagnosis and analyse the status of inflammation and fibrosis. After surgery, long-term hormonal therapy was offered at the hospital to all women not trying to become pregnant.

The primary outcome was the confirmation of diagnosis, a change in pain symptoms, quality of life assessment and pregnancy in cases of patients who wanted children at the follow-up visit. A therapeutic response defined a > 50% reduction in symptoms. Patients who did not visit our outpatient clinic were contacted by telephone at least three times.

### Data evaluation and statistical analysis

Statistical analysis was performed using IBM SPSS Statistics software, version 26 (IBM Corporation, Armonk, NY, USA). If data are missing, the total number of cases with available information is referred to. Categorical variables are reported as frequencies and percentages. Continuous variables are reported as the mean and standard deviation. Spearman’s correlation coefficient was used to compare non-normally distributed variables. A within-group comparison was undertaken with the Wilcoxon rank-sum test for nonparametric data. We performed a stepwise backward logistic regression to assess potential clinical characteristics independently associated with pain scores. A value of *p* < 0.05 was considered statistically significant.

## Results

A total of 94 patients, who showed symptoms resistant to medical treatment and had received peritonectomy within a time period of 5 years at our endometriosis center, were included in this study. The follow-up data of 82 patients (87.23%) were available.

The average age of the patients was 29.40 years (± 6.751) at the time of surgery, 84.9% of women were nulliparous, 15.1% were uni- or multiparous. 42.6% completed a preoperative questionnaire, while the other patients were questioned in detail during a personal interview.

The vast majority (91.2%) of women had taken pain medication (non-steroidal anti-inflammatories or spasmolytics) before surgery without sufficient pain relief, and 68.1% women had taken at least one form of hormonal treatment (combined oral contraceptives, progesterone only pills or a contraceptive vaginal ring) (Table 2). Hormonal treatment was interrupted at least 2 months prior to surgery in all cases.

**Table 2:** Characteristics of the patients included

Age (years) ± SD	29.40 ± 6.751
Nulliparous ^*86^	73 (84.9%)
Multiparous ^*86^	13 (15.1%)
Pain killer preop. ^*57^	52 (91.2%)
Hormonal treatment preop. ^*91^	62 (68.1%)
Vegetative symptoms ^*64^	38 (59.4%)
Nicotine ^*38^	13 (34.2%)
Depression ^*88^	15 (17%)
Adenomyosis sonographically ^*82^	62 (75,6%)
Normal pelvic situs ^*82^	15 (18.3%)
Other pathological finding ^*82^	5 (6.1%)
First surgery	47 (50%)
Surgery for other reasons (such as appendectomy, ovarian cysts and emergency diagnostic laparoscopy)	6 (6.4%)
Previous surgery for EM	41 (43.6%)
EM diagnosed preop. ^*93^	49 (52.7%)
Child wish ^*93^	61 (65.6%)
Impaired fertility ^*61^	29 (47.5%)

*n = number of women included in this subanalysis, variation due to missing data

The majority of women had vegetative symptoms (59.4%), such as nausea, vomiting, headache, migraine, diarrhea and obstipation. About one-third (34.2%) indicated nicotine abuse while nearly one-fifth (17%) suffered from depression (Table 2).

Primary surgical treatment was performed in one-half of cases. 43.6% of patients had previous abdominal surgical interventions for endometriosis. Accordingly, in about one-half of the patients’ endometriosis was diagnosed preoperatively.

### Primary indications

Ultrasound examinations preoperatively revealed adenomyosis in three-quarters of the patients and any other pathological findings, such as the suspicion of extra ovarian cysts or a malformation of the uterus, were found in 6.1%. In 18.3% a normal pelvic situs was diagnosed (Table 2).

86% of patients disclosed that they suffered from dysmenorrhea, 67.4% had cyclic pain, 55.9% reported complex chronic pain, 62.4% dyspareunia, 21.5% dysuria and 35.5% dyschezia. For pain levels please see Table 3.

**Table 3:** Presurgical symptoms

Dysmenorrhea preop. ^*93^	80 (86%)
Cyclic pain preop. ^*92^	62 (67.4%)
CCP preop. ^*93^	52 (55.9%)
Dyspareunia preop. ^*93^	58 (62.4%)
Dysuria preop. ^*93^	20 (21.5%)
Dyschezia preop. ^*93^	33 (35.5%)
Strength of dysmenorrhea preop. average and max. (median)	7 and 9
Strength of cyclic pain preop. average and max. (median)	5 and 7
Strength of chronic pain preop. average and max. (median)	5 and 8
Strength of dyspareunia preop. average and max. (median)	4 and 7
Strength of dysuria preop. average and max. (median)	2 and 6
Strength of dyschezia preop. average and max. (median)	4 and 7

*n = number of women included in this subanalysis, variation due to missing data

65.6% of women said they wanted to have a child preoperatively and impaired fertility was seen in about one-half of these patients (Table 2), out of these three had further problems complicating/aggravating fertility, namely Asherman, PCO and adrenogenital syndrome.

### Intraoperative findings

Endometriosis was clinically confirmed in all cases on the basis of a conspicuous peritoneum. At the time of surgery, almost all women showed stage I or II endometriosis (44.7 and 48.9%, respectively) as classified by the rASRM score. 6.4% indicated stage III, in 17% of patients, in addition to pelvic peritoneal lesions, extragenital endometrial lesions were found outside of the pelvis, mainly on the diaphragm (Table 4). Of the 73 patients who underwent chromopertubation, a bilateral fallopian patency was seen in 76.7% of cases, unilateral patency in 32.9% and no patency was found in 4.1%. There were no complications reported for the duration of the whole study. Residua of endometriosis had to be left for various reasons in seven women: missing informed consent in case of unexpected incidental endometriosis of the diaphragm (two times) and superficial lesions on the large intestine (three cases), which were coagulated, or unsuspected deep infiltrating endometriosis on the sigma (two times).

**Table 4:** Intraoperative findings

Tubal patency	73 (87.7%)
No patency ^*73^	3 (4.1%)
Patency both sides ^*73^	56 (76.7)
Patency one side ^*73^	24 (32.9)
rARSM I	42 (44.7)
rARSM II	46 (48.9%)
rARSM III	6 (6.4%)
EM genitalis externa	16 (17%)
Histological diagnosis: EM ^*90^	25 (27.8%)
Histological diagnosis: EM and inflammation ^*90^	54 (60%)
Histological diagnosis: inflammation ^*90^	11 (12.2%)

*n = number of women included in this subanalysis, variation due to missing data

**Fig. 1:**
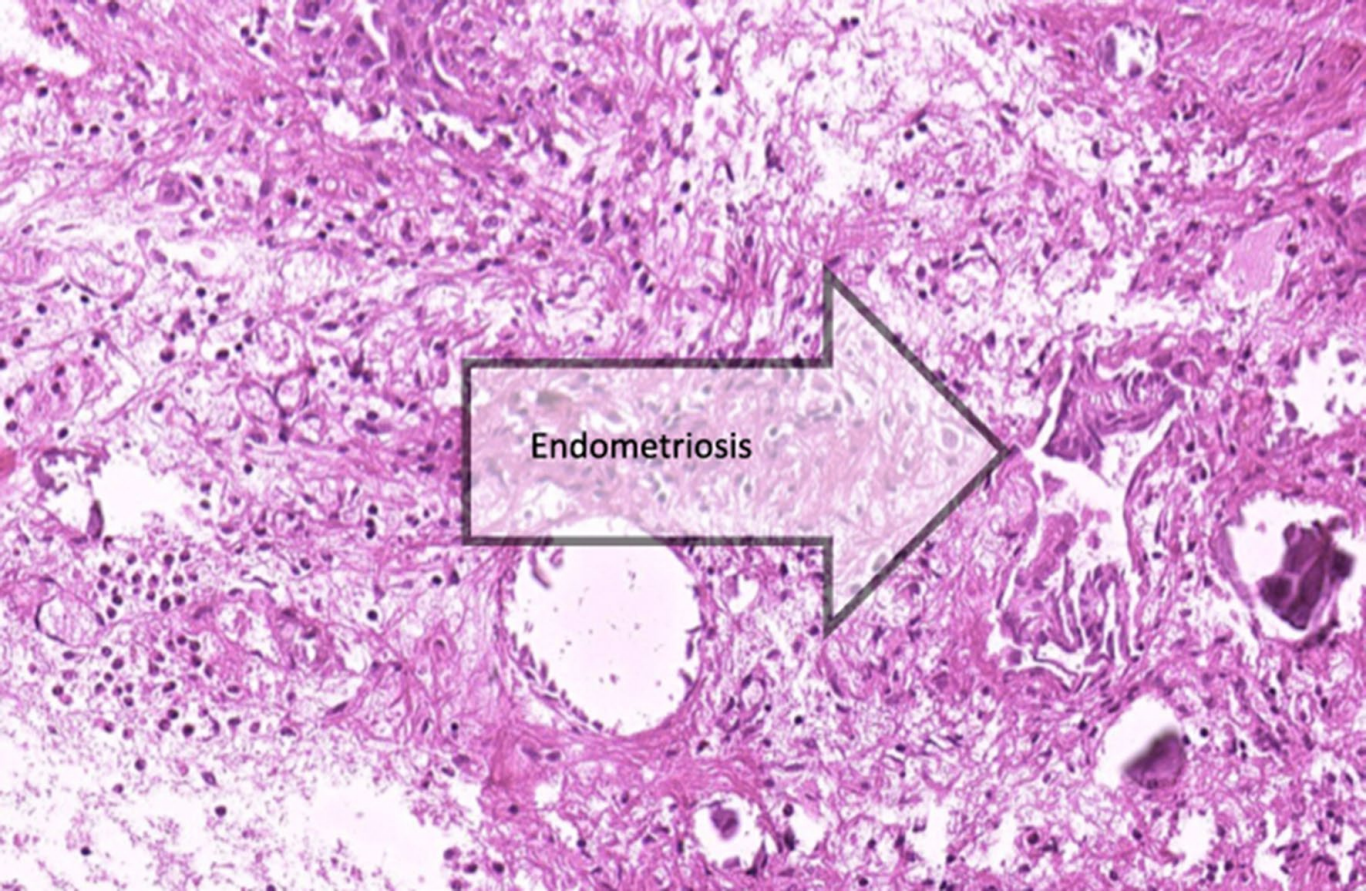
some acute and chronic inflammatory cells, psammomatous calcification

88% had histologically confirmed endometriosis combined with chronic inflammation, and fibrosis, and the remaining patients had signs of peritoneal inflammation/fibrosis only (Fig. 1).

### Post-operative outcome

The mean time after which the follow-up questionnaire was completed was 14.86 months (± 12.792, range 2–59 months).

**Fig. 2:**
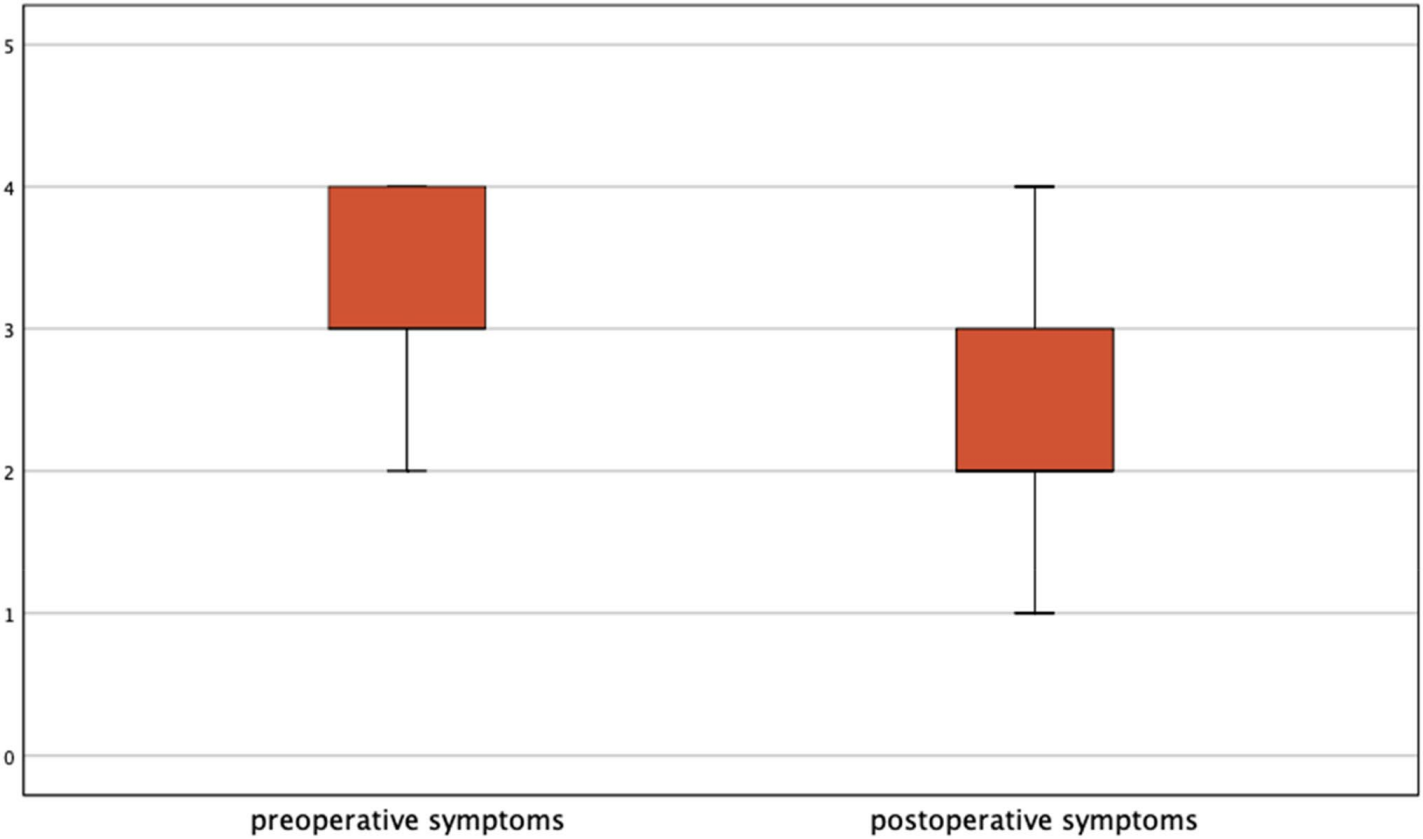
Significant difference between symptoms before and after surgery presented by box-plots. Pain score divided into 4 categories: 4: severe (VAS 8–10), 3: modeste (VAS 5–7), 2: mild (3–4), 1: no pain

**Fig. 3:**
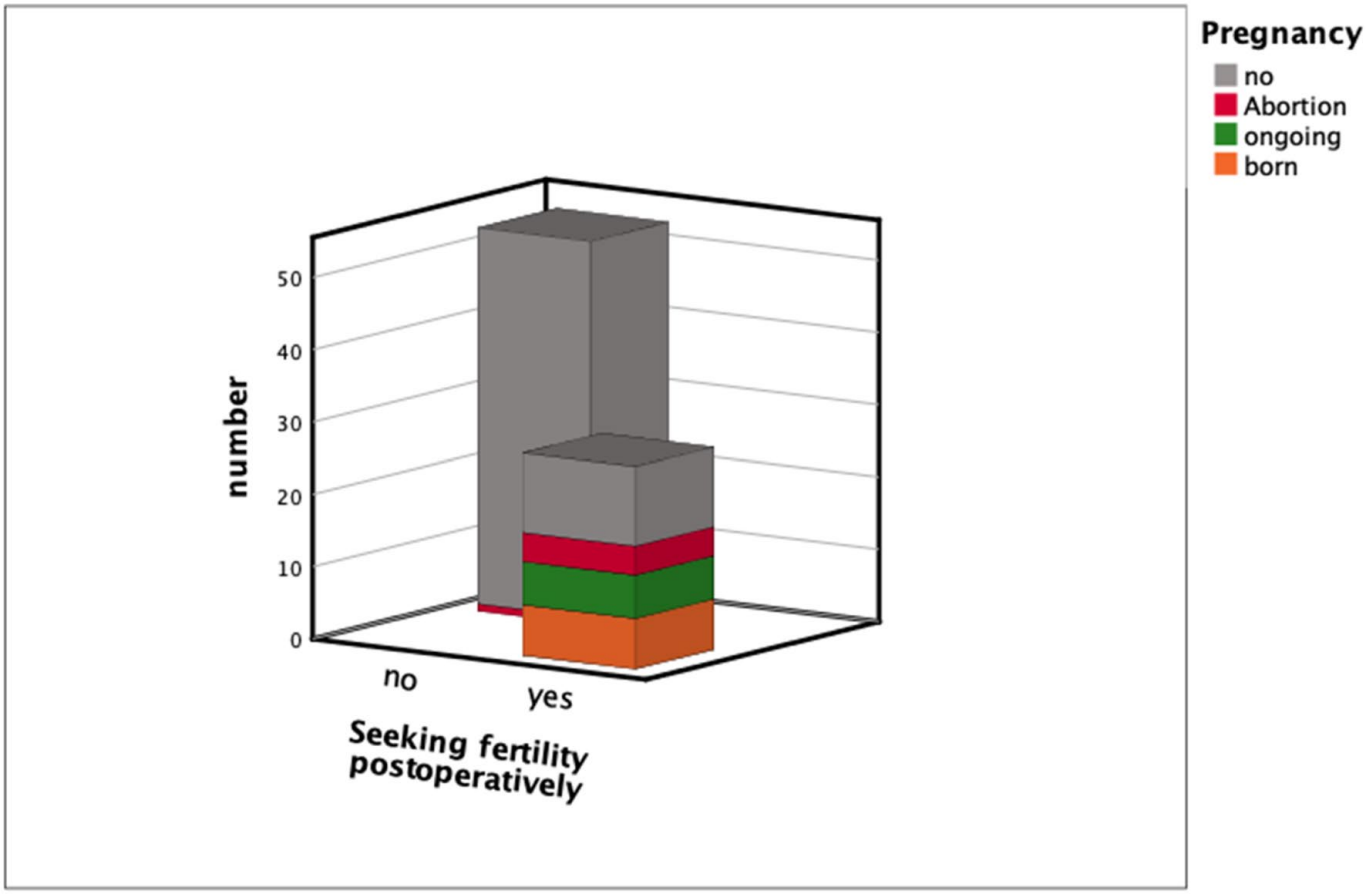
Fertility rate after surgery

Postoperative data showed a remarkable improvement in the quality of life of the majority of patients. More than three-quarters of women reported pain relief (Fig. 2). Of these patients, 23.8% reported symptom-free status and in 52.5% of the women endometriosis-associated symptoms improved greatly. Significant results were reported postoperatively in fertility rates. Within the specified timeframe between performed surgeries to follow-up, 62.07% (18/29) of women with infertility problems became pregnant post surgically, seven women had already delivered, six women had an ongoing pregnancy, four patients had an abortion, one patient had a biochemical pregnancy (Fig. 3). Only three out of these 18 women needed assisted reproductive technology (ART).

Despite our insistent recommendation only 29.87% (23/77) of patients took postoperative hormonal treatment (HT), 11 women rejected the hormonal therapy, seven women did not tolerate therapy, in one patient the gynecologist refused to prescribe HT. In six women multimodal pain therapy was proposed. Twenty-nine patients wanted to get pregnant and consequently opted against HT (Table 5).

**Table 5:** Follow-up

Time of follow-up (range)	14.86 ± 12.792 (2–59)
Re-operation ^*83^	7 (8.4%)
No pain after surgery ^*80^	19 (23.8%)
Symptoms improved after surgery ^*80^	42 (52.5%)
Symptoms unchanged/worse after surgery ^*80^	19 (23.8%)
Child wish post surgically ^*81^	29 (35.80)
Deliveries ^*29^	7
Ongoing pregnancies ^*29^	6
Abortion ^*29^	5
ART ^*18^	3 (16.7%)
Hormonal therapy (HT) after surgery ^*77^	23 (29.87%)
HT rejected ^*77^	11 (14.29%)
HT not tolerated ^*77^	7 (9.09%)
Multimodal pain therapy ^*77^	6 (7.8%)

*n = number of women included in this subanalysis, variation due to missing data

**Fig. 4:**
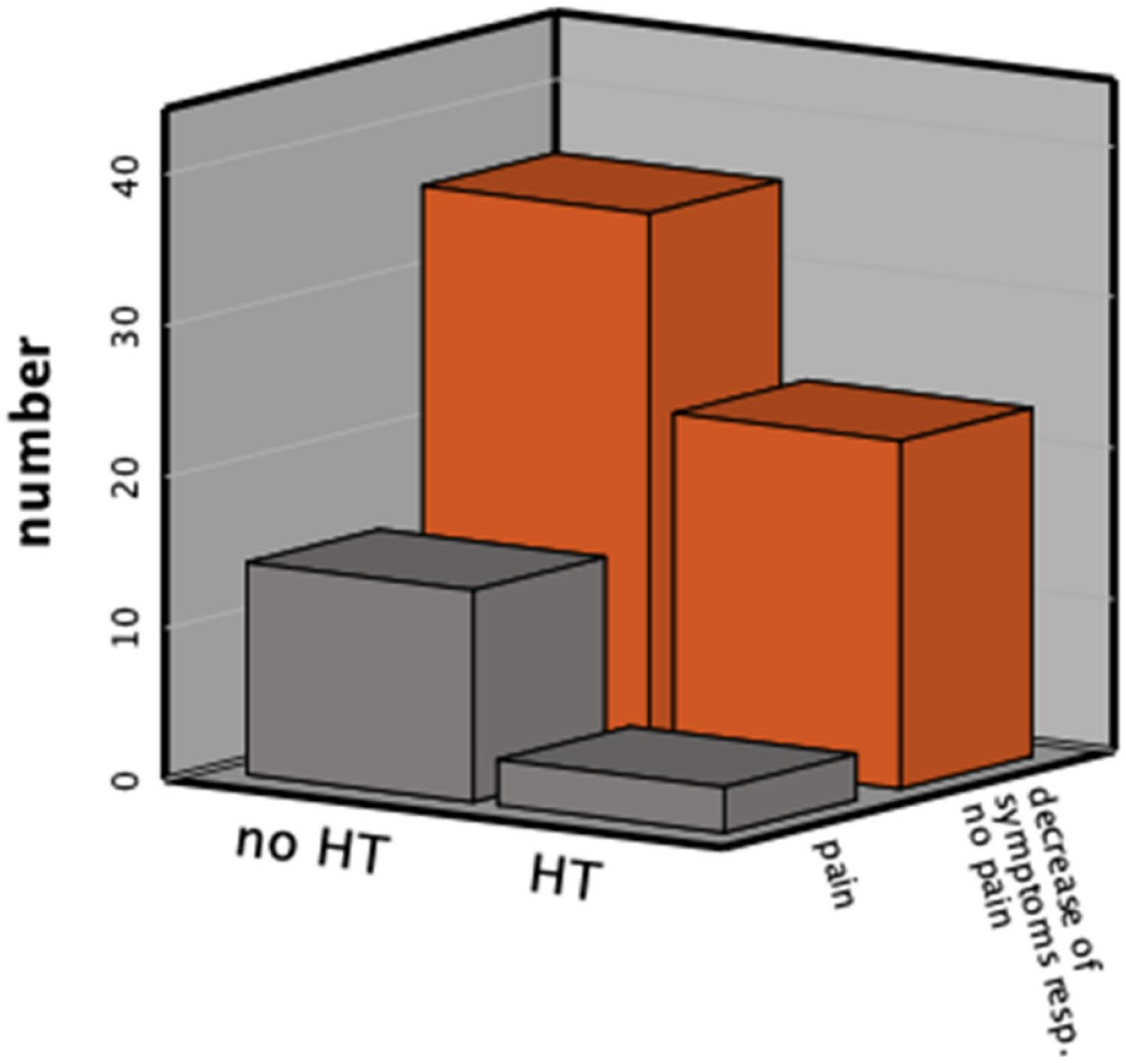
Symptoms post surgically according to hormonal therapy

**Fig. 5:**
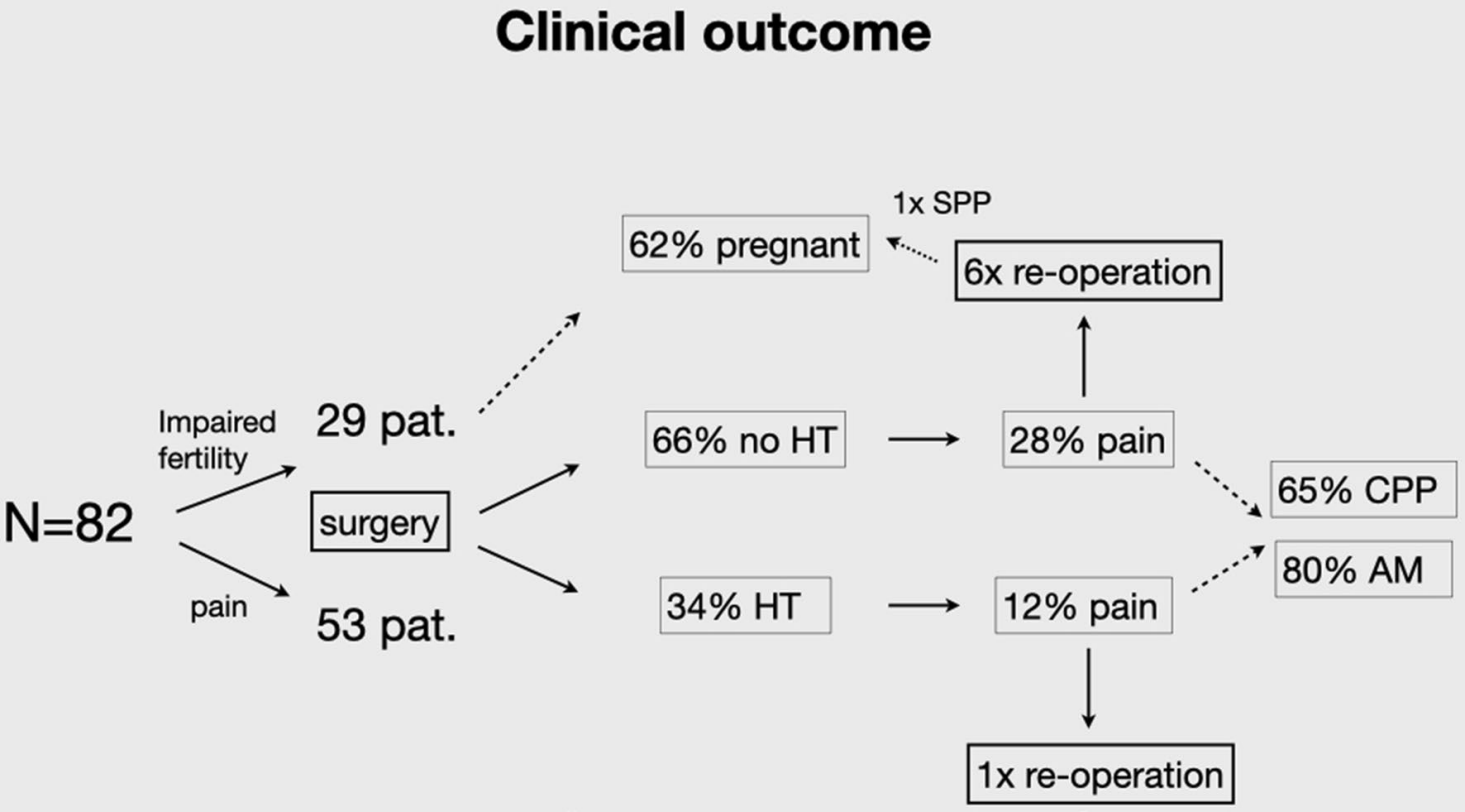
Description of cohort of patients. *HT* hormonal therapy

Symptoms decreased significantly after surgery in the majority of patients (*Z* − 4.330, *p* < 0.000). In comparison to the group of women without HT post surgically, there was a significant decrease of pain in the HT-group (Figs. 4, 5). After analyzing the effect of pre-operation on the outcome, there were no significant differences between the two groups. In seven women a re-operation was performed (see Table 6). The evidence of recurrence was confirmed in only two patients, one of which wished to conceive. This patient underwent fertility treatment for the last 2 years and due to the progression of symptoms and the status of the fallopian tubes we decided to do laparoscopy again. She became pregnant (biochemical pregnancy) after our second intervention. The other patient rejected post-surgical hormonal treatment. The main problem facing the other women was severe adenomyosis. At re-operation, we could see the intact/regrown peritoneum without any signs of inflammation (Fig. 5). We did not detect an excessive presence of adhesions.

**Table 6:** Recurrence

	Surgery before our surgery?	Time interval between surgeries	Indication	Surgery	Intraop. findings	Postop. therapy	Outcome
Case 1	No	2 years	Persistent pain, increased tension of pelvic floor	2nd: diagnostic (performed in another hospital), 3rd: peritonectomy	No EM at 2nd surgery. rARSM I and AM at 3rd surgery	Multimodal pain therapy, HT after 1st surgery. Fertility treatment (2 × IVF) after 2nd surgery	Biochemical pregnancy after 3rd surgery
Case 2	1 year before our surgery, not clear if HT	2 years	Cyclic pain, dysmenorrhea	Peritonectomy	rARSM II	Rejects HT, takes cortisone therapy	1 year after surgery again incipient neurogenic pain
Case 3	No	1 1/2 years	Upon request (to prevent HT)	Diagnostic, removal of isthmocele	Severe AM, no EM. Ovula nabothii in isthmocele	HT	1 year after surgery again incipient cyclic pain (because of retroperitoneal fibrosis?)
Case 4	7 years before our first op, not clear if HT	2 years	Emergency for acute pain	Diagnostic	AM, no EM	HT	After hysterectomy (performed in another hospital) now improvement of symptoms
Case 5	1 year before our first op, long-term treatment with IUD	1/2 year	Upon request	Hysterectomy	Severe AM, no EM	No therapy	Symptoms improved after hysterectomy
Case 6	no	4 1/2 years	Persistent pain, spinal hyperalgesia	Diagnostic, IUD	Severe AM, no EM	Rejects therapy	Asks for hysterectomy
Case 7	1 year before our surgery, not clear if HT	2 years	Upon request	Diagnostic	No EM	HT	

Correlation analysis and logistic regression revealed no effect on the influential parameters of the pain scores and postoperative symptoms.

## Discussion

In summary, endometriosis was clinically confirmed in all cases and laparoscopic excision in our cohort was beneficial in reducing pain, thus improving the patient’s quality of life and enhancing the chance of pregnancy of women in the early stages of endometriosis for more than 1 year following the check-up. However, to achieve these objectives, a well-considered selection for surgery in endometriosis patients is crucial and adequate timing guarantees the highest benefit [50]. Endometriosis has to be understood as a chronic disease which needs individual concepts. Especially the first surgery has to be planned and performed very carefully [51]. Early and recurrent surgeries due to inadequate evidence have to be avoided, as it is well known that endometriosis patients generally need multiple surgeries and have a poor physical and mental health status, and there is a higher chance that disease recurrence happens [52].

The success rates for reducing the characteristic symptoms of endometriosis have been stated in the literature and are similar to the results we found in our study. Almost 25% were non-responders in a recent study by Ghai et al. on 102 women with superficial endometriosis, independent on the surgical method (excision or ablation) [53]. Interestingly, women were more likely to be non-responders if treated for early-stage endometriosis compared with those with severe endometriosis. One reason for this might be that surgeries are often done under hormonal treatment like combined oral contraception, so the extent of peritoneal endometriosis is underestimated and implants are left. One study by Strowitzki et al. clearly showed a downstaging of peritoneal endometriosis under hormonal treatment with dienogest [54]. However, data on presurgical suspended hormonal therapy are usually missing.

The pelvic peritoneum appears to play a key role in the development and maintenance of endometriosis. The attempt of hormonal therapy before surgery helps to identify patients with exclusively acyclic pain resistant to hormonal treatment as an indication for the occurrence of peritoneal lesions with neurogenic inflammation. Such patients might have a benefit from the excision of these lesions. Accordingly, it was shown that the removal of peritoneal lesions remarkably decreased not only the pain level, but the low pain threshold went back to the normal level of healthy controls [55–57]. Another benefit of excision is the histologic confirmation of the disease. Nearly 100% of our patients had an altered peritoneum with a histologically proven disease and/or inflammation. Histologic diagnosis is, however, dependent on pathologist’s experience [58].

The patients’ hormonal therapy had been stopped before surgery (minimum 2 months prior to surgery) due to the chance of failure. So, the altered peritoneum was more visible and glassy lesions and inflammation could be removed. Interestingly not only the endometriotic lesions could be confirmed, but also the presence of inflammation in nearly 75% of cases. This extended inflammatory reaction might be underestimated as an essential part of pain generation. However, certain patients suffered from persistent pelvic pain after the excision of endometriosis. This might also be associated with adenomyosis, a main cause of dysmenorrhea. During sonography examinations, it was discovered that three-quarters of women in our cohort had adenomyosis. It is well known that in up to 90% of the cases, endometriosis and adenomyosis appear at the same time [59].

In more than 20–25% of patients, pain still remains a part of their daily life despite the well processed surgical or hormonal treatment [60]. Similarly, in our cohort 23.7% of patients experienced no benefit after surgery (Table 5). However, 11 of them (57.9%) rejected adjuvant hormonal treatment.

Our renewed surgery rate is low (8.54%) compared to the probability of a further surgical procedure of about 15–20% according to literature and this may be attributed to the correct suspicion of peritoneal endometriosis and the adequate excision of all areas of abnormal peritoneum (peritoneal lesions and inflamed altered tissue without hormonal downregulation) with a sufficient safety margin in all cases [61–63]. An earlier report demonstrated that one-quarter of the patients with proven peritoneal endometriosis already had microscopic endometriotic implants in their peritoneum that were otherwise deemed normal [64]. And others showed recurrent endometriotic lesions especially in the margin of earlier resection areas [65]. We recommend hormonal treatment following surgery to all our patients with the aim to prevent recurrence [66, 67].

In patients who received further surgery after extensive peritonectomy, all but two had no evidence of endometriosis, neither macroscopically nor histologically at the time of re-operation. This does not signify that endometriosis has not been the cause of pelvic pain but proves the concept of chronic long-term pain which may be a consequence of the up-regulation of pain sensitization and not recurrent disease. Such pain may not go away even after hormonal and/or surgical therapy [68]. These chronic pain patients suffer from spinal hyperalgesia, myofascial pain syndrome or pelvic floor muscle imbalance. Chronic pain is an interplay of pathophysiological, psychological and social factors. The complexity of pain sensation and perception have to be addressed. We recommend multifaceted care models including pain management programs, nutrition advice, counseling and education, osteopathy, and psychological therapies alongside gynecologic treatments to affected women [69].

**Fig. 6:**
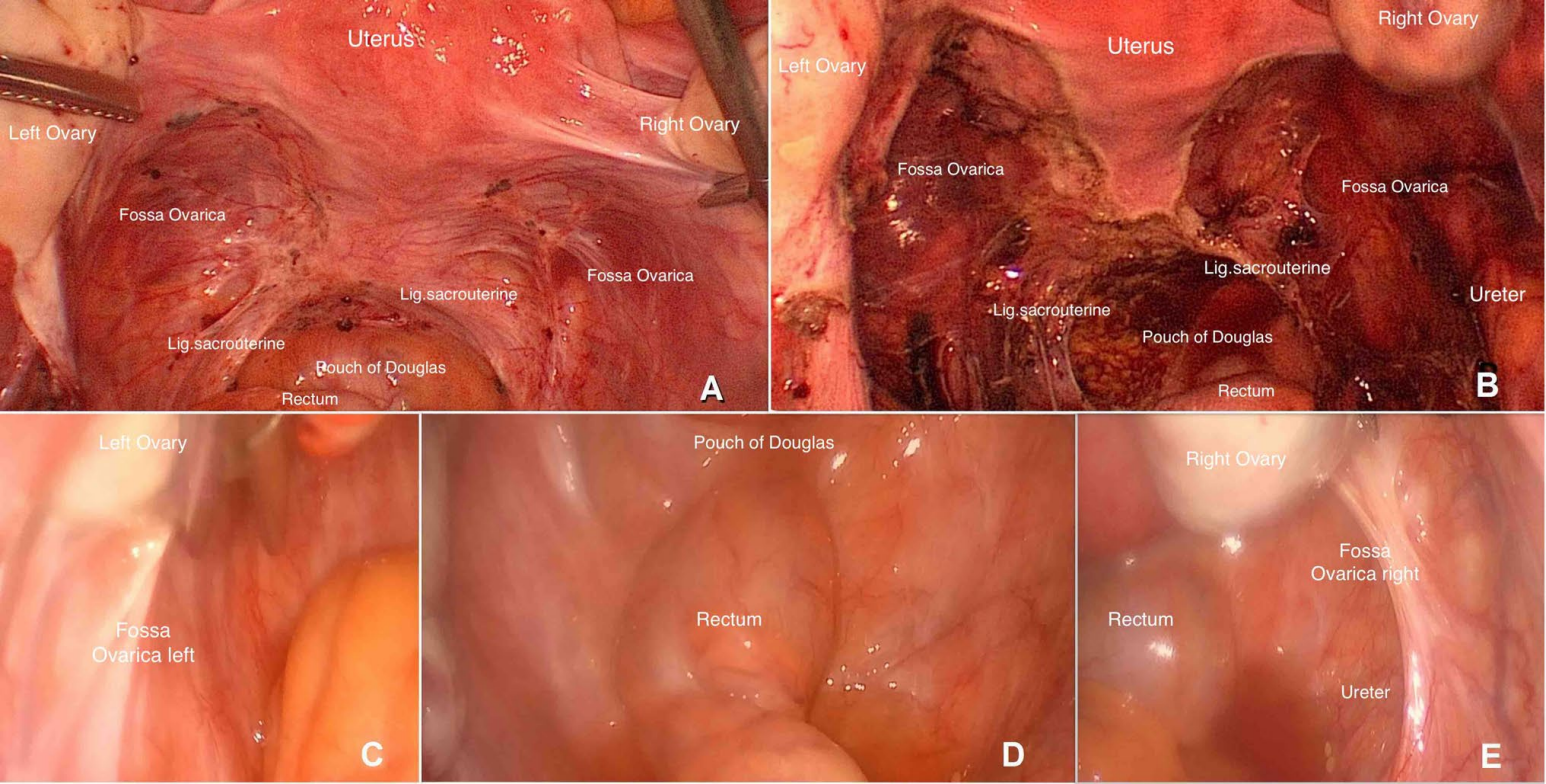
Surgical site before surgery (**a**), after surgery (**b**) and at re-operation (**c**–**e**)

Based on two older contradictory studies and a Cochrane review comparing laparoscopic surgical treatment with diagnostic laparoscopy only in minimal and mild endometriosis, the laparoscopic surgery had better results for pregnancy after 20 weeks, regardless of the surgical method [29–31]. The odds ratio of 1.65 and the number needed to treat of 12 are though viewed critically [61]. In a comparison of the basic chance of pregnancy of about 20% our pregnancy rate is pleasantly more than three times higher. In our view, the surgical removal of peritoneal implants with a safety margin address the nociceptive as well as the neurogenic inflammatory pathway of pain caused by endometriosis. Maybe the excision of the inflamed tissue affects fertility. We recommend timely and comprehensive surgical management and determined fertility treatment in patients wishing to conceive considering the higher chance of conception within 2 years of surgery and the negative impact of repeated surgery on fertility outcomes [50] (see Fig. 6).

### Strengths

One methodological strength of this study is the standardized documentation of clinical data and pain history on a dedicated questionnaire in the majority of cases. Surgery was done with the expectation of peritoneal endometriotic lesions and good and uniform preparation of the patients. All interventions were done by a single high-volume minimally invasive gynecologic surgeon with a focus on endometriosis. Consistent techniques were performed throughout the duration of the study. All patients had to discontinue their choice of standard medical suppression treatment for endometriosis at least 2 months before surgery. All women were evaluated and treated by physicians with long-standing and extensive expertise in the management of endometriosis. This is indirectly confirmed by the observation that endometriosis was confirmed in all cases. In addition, the follow-up interviews were performed mainly face-to-face, reducing the risk of recall bias. Our follow-up rate was high compared to other studies (61.61% in Yeung et al., 60.3% in Riley et al. [70, 71]).

### Limits

**Fig. 7:**
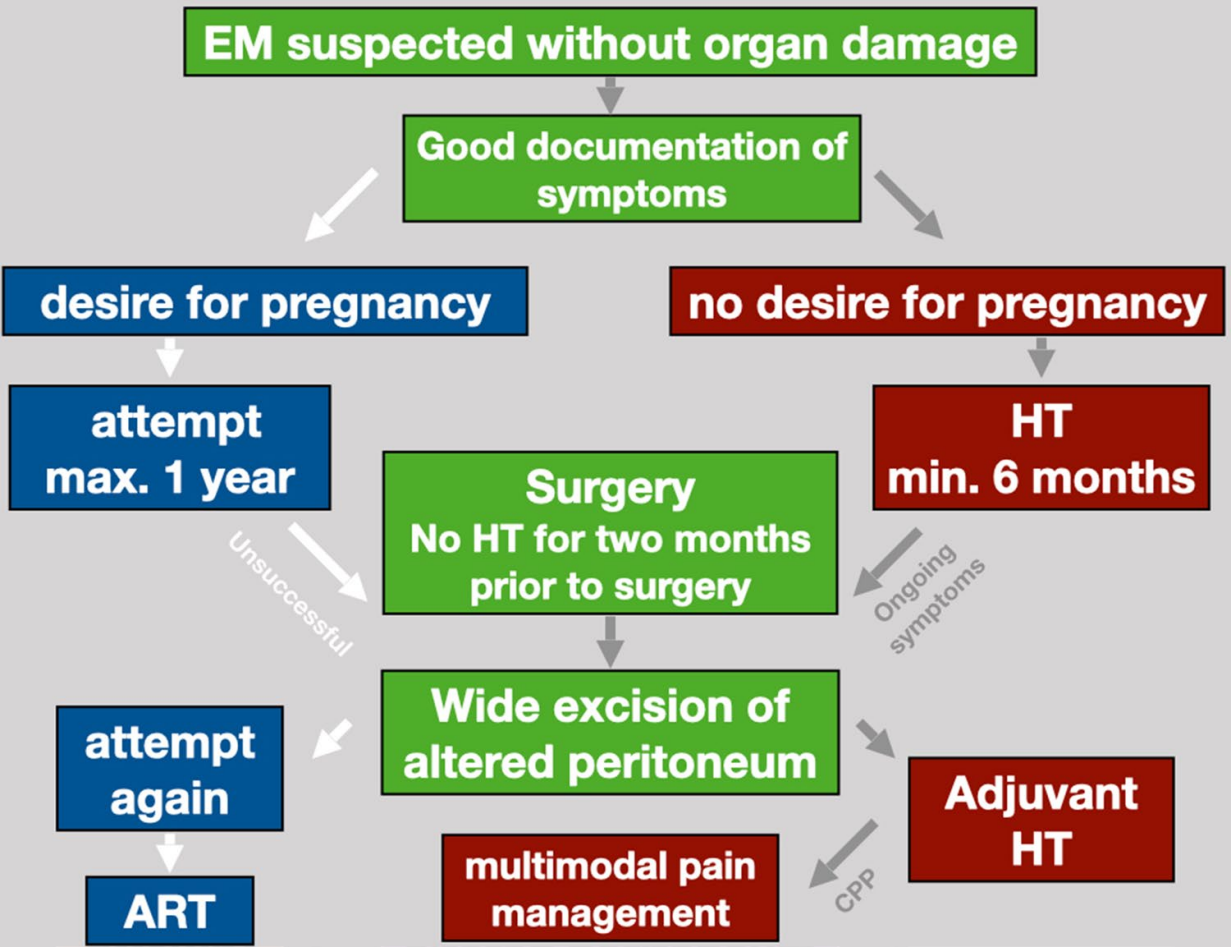
Standard of procedure in our hospital

Regarding the limitations of this study, many women (43.6%) had previously been operated on due to symptoms of endometriosis, prior to having surgical excision. It is possible that this may bias the outcome, with women being preselected because they had had a previously failed therapy. This highlights two points: the first is that the perfect treatment has yet to be found. All current available treatments have a significant “failure rate” as noted by the recurrence of pain and the desire for further treatment. The second is that endometriosis is a very individual chronic disease requiring different treatments depending on the patient’s particular phase of life. Interestingly our surgeries on women, who had been operated previously, were as successful as first time interventions. One possible explanation could be that the first operations had been performed under hormonal therapy (Fig. 7).

The design was one cohort and the time of follow-up was inconsistent. With the given sample size of the study, it cannot be excluded that some of the observed effects could be clouded by subsequent medical or surgical treatment not reported by patients or recorded in our medical files.

We do not have the post-surgical VAS scores of symptoms. An important change for the patient may be one that represents a meaningful reduction in symptoms or improvement in HRQoL from her point of view. Vincent et al. suggest that the definition of a responder in endometriosis corresponds to a > 30 or > 50% reduction in symptoms [72]. We classified trial participants as a responder who called themselves a responder by having had a subjectively satisfying response to therapy (> 50% reduction in symptoms).

It is possible that the results of this study may be affected by the 12.77% of women who were not reached for follow-up, since subjects lost to follow-up notoriously have a worse prognosis [43]. We compared responders with non-responders. Based on this analysis it is unlikely that the results would be significantly altered by women who were not included in the follow-up cohort.

## Summary

We understand endometriosis as a complex and multifactorial disease. Patients with endometriosis need individual management of the disease regarding the personal situation (symptoms and family planning). Early and recurrent surgeries for diagnosis only without any therapeutical concept have to be avoided [50, 51]. Long-term treatment with hormones and multimodal concepts are needed.

## Data Availability

Data will be available upon request.
